# Comparison of the Antifungal Efficacy of 1.3% NaOCl/MTAD with Other Routine Irrigants: An *Ex-Vivo* Study

**DOI:** 10.1155/2014/575748

**Published:** 2014-10-29

**Authors:** Neha Juneja, Mithra N. Hegde

**Affiliations:** ^1^Conservative Dentistry & Endodontics, Manav Rachna Dental College, Manav Rachna Educational Institutions Aravalli Campus, Sector 43, Delhi-Surajkund Road, Faridabad, Haryana 121004, India; ^2^Conservative Dentistry & Endodontics, Department of Conservative Dentistry & Endodontics, A. B. Shetty Memorial Institute of Dental Sciences, Medical Sciences Complex, Deralakatte, Mangalore, Karnataka 575018, India

## Abstract

*Objectives.* To evaluate and compare the antifungal efficacy of 1.3% NaOCl/MTAD with 2.5% sodium hypochlorite (NaOCl), 2% chlorhexidine gluconate (CHX), and iodine potassium iodide (IKI). *Materials and Methods*. Fifty-two single rooted teeth were used which were divided into four groups with 10 teeth in each group: 2.5% NaOCl, 2% CHX, IKI, 1.3% NaOCl/MTAD, and physiologic saline. Two teeth served as negative controls and were placed in fresh brain-heart infusion broth (BHI) after autoclaving. The teeth were inoculated and incubated with *Candida albicans* after which the teeth were instrumented and irrigated with the test irrigants. The first microbial sampling was then performed and colony forming unit/mL (cfu/mL) was counted. The second microbial sampling was performed 1 week after instrumentation and irrigation. *Results*. The test irrigants were effective against *C. albicans* in both the first and second microbial samplings. When the irrigants were compared, there was no statistical difference in their activity in the 1st and 2nd microbial sampling. On comparison of the change in mean cfu/mL between the 1st and 2nd microbial samplings, the antifungal activity of the test irrigants was in the order 2.5% NaOCl > 2% CHX > 1.3% NaOCl/MTAD > IKI.

## 1. Introduction

Infection of the root canals with bacteria causes pulpitis and leads to the initiation and progression of periapical periodontitis. The obvious objective of endodontic treatment is thus to prevent or eliminate infection within the root canal [1].

Mechanical instrumentation for root canal debridement may leave certain areas of the root canal system untouched due to its complex anatomy. It has been shown that mechanical instrumentation without irrigation reduces but does not predictably eliminate microorganisms in the canal. Thus, a root canal irrigant is needed to achieve the goal of complete debridement of the canals [2].

In addition to the identification of bacterial strains, fungi have also been isolated from root canal infections, with the most common isolate being* C. albicans* [3].

In current practice, the most common irrigants used are sodium hypochlorite (NaOCl), chlorhexidine gluconate (CHX), and Iodine potassium iodide (IKI). Biopure MTAD cleanser (Dentsply/Tulsa) is a new root canal cleanser, introduced by Mahmoud Torabinejad and associates in the year 2003. MTAD combines a surfactant (0.5% Tween 80), an acid (4.25% citric acid), and a broad spectrum antibiotic (3% doxycycline) [4].

Studies on the effectiveness of NaOCl, CHX, and IKI against* C. albicans* show these irrigants to be useful in killing* C. albicans*. Waltimo et al. found in their study that NaOCl and IKI killed all the* C. albicans* within 30 seconds. They also tested CHX which they found to take 5 minutes to kill all the yeast cells [3]. Ruff et al. found 6% NaOCl and 2% CHX to be equally effective and significantly superior to Biopure MTAD in their antifungal activity [5]. However, there is still not sufficient evidence on the antifungal activity of Biopure MTAD.

Thus, the present study was conducted with the aim to evaluate the antifungal activity of MTAD and also to compare its action with that of routine irrigants, namely, 2.5% NaOCl and 2% CHX and IKI.

## 2. Materials and Methods

Clearance from the institutional ethical committee was obtained prior to the conduct of the study. Fifty-two freshly extracted single rooted human teeth (excluding mandibular anterior) were used in the study. The teeth were collected, stored, disinfected, and handled as per the recommendations and guidelines laid down by OSHA and CDC. Teeth with open apices, resorptive defects, cracks on root surface, and gross caries involving the crown or root, exceptionally thin and curved roots, and teeth with more than one root canal were excluded. All teeth were radiographed from facial and proximal orientation to confirm the presence of a single canal.

### 2.1. Test Organisms

The microorganism used in this study was* Candida albicans* which was obtained from the Department of Microbiology, K. S. Hegde Medical Academy, Mangalore.* Candida albicans* was previously cultivated in Sabouraud's dextrose agar (SDA) broth (HiMedia Lab, Mumbai, India) for 48 hrs and then cultured on SDA plates. The SDA plates were then incubated aerobically at 37 ± 1°C for 48 hrs. Suspensions of* C. albicans* had the optical density adjusted spectrophotometrically to approximately 1.5 × 10^8^ cfu/mL.

### 2.2. Irrigants

Commercial preparations of Biopure MTAD (Dentsply Tulsa Dental, Johnson City, TN) and 2% chlorhexidine gluconate (Dentaclor, Ammdent, Chandigarh, India) were obtained.

The 2.5% NaOCl was freshly prepared from 4% NaOCl (Fischer Scientific, Mumbai, India). Iodine potassium iodide was freshly prepared by dissolving 2% iodine (Merck, Mumbai, India) in a 4% aqueous solution of potassium iodide (Rankem, Delhi, India).

Biopure MTAD was used with 1.3% NaOCl as per the manufacturer's instructions. We prepared fresh solutions of 1.3% NaOCl from 4% NaOCl (Fischer Scientific, Mumbai, India).

### 2.3. Initial Preparation of Root Canals

The crowns of all the teeth were sectioned with a diamond disk (Novo Dental Products, India) and the root lengths were standardized to approximately 16 mm. They were then stored in physiologic saline solution (Baxter India Pvt. Ltd., Alathur, India) until use.

Root canals were instrumented 0.5 mm beyond the apical foramen up to size 25 K file (Mani Inc., Tochigi, Japan). Root canals were then instrumented 1 mm short of the root apex up to size 40 K files (Mani Inc., Tochigi, Japan). Irrigation was performed with 1 mL of physiologic saline solution between each instrument. The root surfaces were then coated with an epoxy resin, except the cervical access and apical foramen.

After setting the epoxy resin, the root canals were filled with 17% EDTA (Ammdent, Chandigarh, India) for 3 minutes in order to remove the smear layer. This was followed by 5 mL of physiologic saline solution.

### 2.4. Grouping of the Specimens

**Figure 1:**
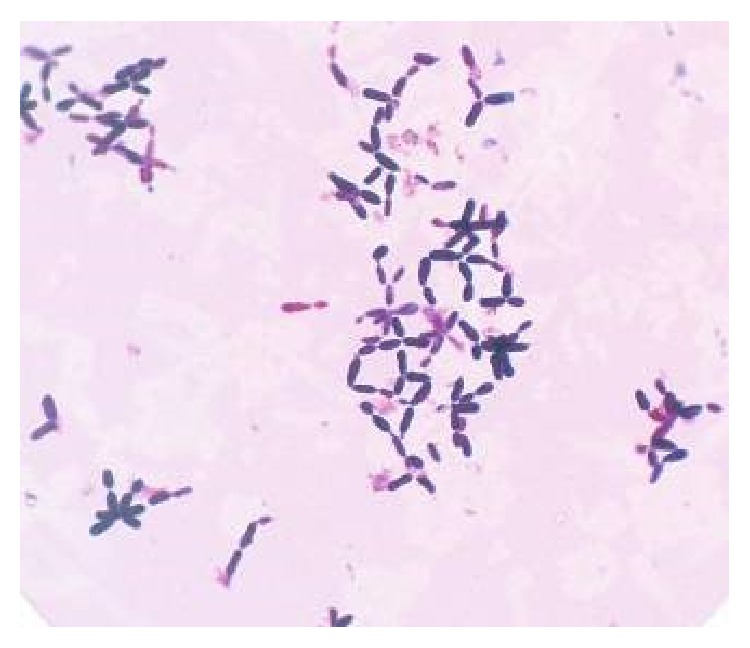
Candidal hyphae stained positive with Gram staining (x40).

Root apices of 50 teeth were sealed with temporary cement (Cavit, 3MESPE). For the convenience of instrumentation and irrigation, these teeth were mounted in dental stone casts with 10 teeth per cast. All the casts and the 2 teeth for the negative control were then autoclaved. All the procedures following autoclaving were performed in a Laminar flow. Two teeth were then placed in sterile BHI broth (negative control). The five casts were randomly divided into subgroups A1, A2, A3, A4, and A5 and were contaminated with 30 *μ*L of* C. albicans* suspension using a micropipette. A sterile cotton ball was soaked in a suspension of microorganisms and placed in the cervical third of the canal. Afterwards, the cervical access was sealed with temporary cement. The roots were then incubated at 37 ± 1°C for 7 days. Each day, the temporary cement was removed and fresh SDA broth was added, with an insulin syringe of 0.5 mL. Throughout the period of the study, the stone casts were kept partially immersed in distilled water in a sterile stainless steel tray with a closed lid to maintain humidity required for the growth of the organisms. To confirm infection, samples were taken with paper points from a randomly chosen specimen from each group on the 5th day and plated on to Sabouraud's dextrose agar and incubated for 48 hrs. The colonies that developed after 48 hrs were verified to be the tested organism by the colony morphology and by doing a gram staining [5] (Figure 1).

### 2.5. Placement of Irrigants

After contamination, teeth in groups A1, A2, A3, A4, and A5 were irrigated with 2.5% NaOCl, 1.3% NaOCl and Biopure MTAD, 2/4% iodine potassium iodide, and 2% chlorhexidine gluconate and physiologic saline, respectively.

The procedure in each group can be summarized as follows.

The teeth were instrumented with size 45 and size 50 K files using 1 mL of the corresponding test irrigant after each file. After instrumentation, each canal was flushed with 5 mL of the test irrigant which was left in the canal for 5 minutes. After 5 minutes, the canals were irrigated with 5 mL of physiologic saline to wash away the test irrigant. A total volume of 7 mL of the test irrigant was used in irrigating each canal. For group A2, the instrumentation with each file was followed by 1.3% of NaOCl which was followed by 1 mL of Biopure MTAD which was allowed to remain in the canal for 5 minutes. After 5 minutes, the canals were flushed with 4 mL of Biopure MTAD according to the manufacturer's instructions. This was followed by 5 mL of physiologic saline.

### 2.6. Incubation and Microbial Sampling

With the canals filled with the saline, the first bacteriological sampling was taken using a sterile paper point size 50 that was placed inside the root canal for 1 minute. The fluid in the canal was absorbed by the paper point and transferred to a test tube containing 0.5 mL of sterile saline solution and shaken for 30 s. Then 0.1 mL aliquot was plated on SDA. The SDA plates were then incubated aerobically 37 ± 1°C for 48 hrs. Microbial growth was verified and the number of cfu/mL of* C. albicans* was counted and confirmed by Gram stain in a light microscope.

After the first microbial sampling, the roots were filled with SDA broth for* C. albicans* and the cervical access was again sealed with temporary cement and the teeth were incubated for a further 7 days. After this period, the second microbial sampling was performed following the same procedures as the first microbial sampling. Throughout these 7 days, the media were refreshed each day in all the canals.

### 2.7. Statistical Analysis

Results obtained were statistically analyzed using Kruskal Wallis test and Wilcoxon signed rank sum test. Significance was based on *P* values.

## 3. Results

Seven days after inoculation of the microorganisms into the root canals, it was observed by culture and gram staining that all groups showed the growth of* C. albicans*. The negative control showed the absence of turbidity throughout the period of the study.

**Figure 2:**
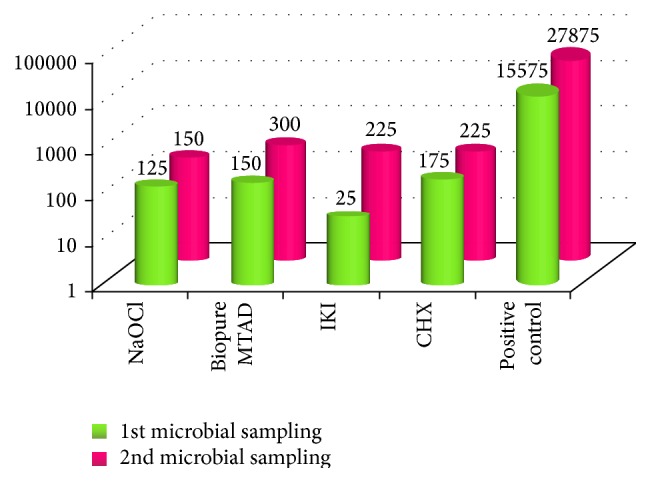
Bar graph showing the difference in the mean cfu/mL of* C. albicans* in the 1st and 2nd microbial sampling between the positive control and the test irrigants.

The growth of* Candida albicans* was significantly less in the test groups in both first (*P* = 0.001, vhs) and second microbial samplings (*P* = 0.001, vhs) as compared to the positive control group (Figure 2). Comparison between the test groups did not show any statistical difference in inhibiting the growth of* Candida albicans* at first (*P* value = 0.266, ns) as well as second microbial sampling (*P* value = 0.419, ns). From Table 1, it can be inferred that group A3 (*P* = 0.039) showed a significant change in cfu/mL between the first and second microbial sampling and was thus less effective than groups A1, A2, and A4 in its antifungal activity. The comparison of the mean cfu/mL between the first and second microbial sampling (Figure 2) highlighted group A1 (2.5% NaOCl) followed by group A4 (2% CHX) to be most effective in the second microbial sampling followed by group A2 (1.3% NaOCl/MTAD) in their long-lasting antifungal effect.

## 4. Discussion

Many studies have shown that microorganisms and their products, byproducts, and toxins are the main cause of the development and persistence of pulpoperiapical lesions [6]. According to the evolution of the pathological process, they are not restricted to the canal lumen, but can reach deeper portions inside the dentinal tubules, root canal system, external root surface (cementum resorption), or even the periapical lesion when present. Therefore, an irrigating solution that possesses antimicrobial activity is required [7].

Studies of the dynamics of root canal infection show that the relative proportions of anaerobic microorganisms and other bacterial cells increase with time and that the number of facultative anaerobic bacteria increases when root canals remain infected for long periods [6, 8]. In the last decade, incidence of* C. albicans* in endodontic infection has received attention and fungi were observed both in primary and refractory endodontic infections. In the last decade, incidence of* C. albicans* in endodontic infection has received attention and fungi were observed both in primary and refractory endodontic infections [9–12]. Among those fungal infections,* C. albicans* was the most frequently found type [9].

Various methodologies can be used to assess the antimicrobial activity of endodontic irrigants. A difference in the methodologies used to assess the antimicrobial activity of irrigants explains the differences found in the results of various studies. Some methodologies allow direct contact of the irrigant with the microorganism (as in the agar diffusion test) while others, such as in our study, may not necessarily allow a direct contact of the irrigant with the microorganism entombed inside the dentinal tubule.

A number of investigators have used infected dentin blocks prepared from bovine teeth to determine the ability of root canal irrigants and medications to disinfect dentin [13]. Despite the similarities between bovine and human dentin, the tubule size of bovine dentine is significantly larger than that found in human teeth. Thus, in our study, we selected human extracted teeth to study the antimicrobial effect of irrigants [14].

Microbiological sampling is another important step that varies among the different methodologies. In this study, the bacteriological sampling was accomplished with a sterile paper point that absorbed the root canal contents. The paper point was then transferred to tubes containing saline solution that were plated in SDA agar plates. The use of paper points has the advantage that it can be performed* in vitro* and* in vivo*. On the other hand, microbiological sampling with paper points is limited because only the microorganisms that are in the root canal can be sampled, while the ones that are located inside the dentinal tubules are not.

In our study, the microorganisms were allowed to grow within the root canals for a period of 7 days for dentin tubule infection. The 7-day incubation period chosen in the present study leads to well infected dentin tubules as has been confirmed by SEM study by Menezes et al. [15]. They showed that both* E. faecalis* and* C. albicans* penetrated well in the dentin tubule after 7 days. In our study, we confirmed the viability of microorganisms by positive culture tests.

Our study aimed at not only investigating the efficacy of irrigants immediately after irrigation but also their residual activity. Past studies [7, 16, 17] have shown that the bacteria that survive the instrumentation and irrigation quickly proliferate and recolonize root canals that remain empty between treatment sessions. Study by Menezes et al. showed that even* Candida albicans* recolonized the root canal after seven days [15]. Thus, we studied the second microbial sampling in all the groups after 7 days of irrigation to assess the residual activity.

The roots were initially instrumented up to size 40 K file to create space for introducing the microbial suspension. Also 17% EDTA was used in order to remove the smear layer created by the instrumentation before the period of contamination in order to allow the penetration of the bacteria and the fungi into the tubules [14]. After the contamination period, the instrumentation from size 40 K file up to size 50 K file was performed to verify the antimicrobial action of irrigants in combination with biomechanical preparation, thus stimulating the clinical situation.

In the present study, 2.5% NaOCl and 2% CHX were used as they are commonly used as irrigants during endodontic treatment [15]. The use of IKI as an endodontic irrigant was suggested by Hancock III et al. [18] Biopure MTAD is a new product that has antimicrobial properties due to the presence of doxycycline. Literature regarding the antifungal efficiency of this irrigant is insufficient.

The negative control showed an absence of turbidity throughout the period of our study, thus demonstrating that the sterilization procedures utilized were effective.

Comparing the action of the irrigants against* C. albicans,* it was found that all the irrigants were significantly effective in reducing its growth. There was however no significant difference in the antifungal activity of these test irrigants. The antifungal activity of 2.5% NaOCl and 2% CHX and IKI as seen in our study is in accordance with the previous studies [3, 5, 15]. However, the efficacy of Biopure MTAD against* C. albicans* as seen in our study is in contrast to the study by Ruff et al. [5]. This can be explained by the use of 1.3% NaOCl with MTAD in our study as compared to the use of only MTAD in their study. Thus, 1.3% NaOCl may be responsible for the antifungal effect seen in our study.

From a comparison of the change in the cfu/mL between the first and second microbial sampling, we also concluded that the antifungal substantivity of IKI was less than the other test irrigants. This short duration of activity of IKI can be explained by the fact that IKI has iodine as its active component which shows a long distance antimicrobial effect through its evaporation and sublimation. Thus, iodine being volatile is responsible for IKI to not have any substantivity. A comparison of the other three test irrigants however leads us to conclude that the antifungal substantivity of 2% CHX and 2.5% NaOCl was greater than 1.3% NaOCl/MTAD. The substantivity of CHX is because of its ability to adsorb to the surfaces covered with acidic proteins, such as hydroxyapatite, and be gradually released in the form of an active cation [19].

Thus, we suggest the use of any of the three irrigants, 2.5% NaOCl, 1.3% NaOCl/Biopure MTAD, and 2% CHX, during biomechanical preparation for their antifungal effect as all were found to be equally effective against the* C. albicans*. However, the use of MTAD provides both a good antimicrobial activity along with smear layer removal [20] as well as tissue dissolution when used with 1.3% NaOCl. Thus, the use of Biopure MTAD may be more preferable especially with the emergence of the monoblock concept and the use of adhesive techniques in the root canal.

The methodology used in this study tried to simulate the clinical situation; however, it is not superior to an* in vivo* study. Thus, further studies with these irrigants* in vivo* are recommended.

Within the limitation of this study, the following conclusions were drawn.

1. All irrigants were effective against* C. albicans* when compared to the positive control in both the 1st and the 2nd microbial sampling.
2. A combination of 1.3% NaOCl/MTAD provides good antifungal activity.
3. IKI was less effective than the other test irrigants in its antifungal effect after seven days.

## Figures and Tables

**Table 1:** Mean change in the cfuMl^−1^ between the 1st and 2nd microbial samplings for the test irrigants and the positive control using Wilcoxon's sign ranked sum test.

Groups	Paired differences		*P*
	Mean	Std. deviation	
Group A			
A1 (2.5% NaOCl)	−25.0000	218.89876	0.705
A2 (1.3% NaOCl/MTAD)	−150.0000	357.46018	0.150
A3^*^ (IKI)	−200	258.19889	0.039
A4 (2% CHX)	−50.0000	437.79752	0.863
A5^*^ (physiologic saline)	−12300.0000	4334.61520	0.005

^*^Significant change in the cfu/mL between the first and the second microbial samplings.
